# Accelerated theta-burst transcranial magnetic stimulation of the bilateral dorsolateral prefrontal cortex in a patient with post-traumatic stress disorder and major depressive disorder: Case report

**DOI:** 10.3389/fpsyt.2023.1061088

**Published:** 2023-02-02

**Authors:** Chun-Hung Chang, Chieh-Yu Liu, Shaw-Ji Chen, Hsin-Chi Tsai

**Affiliations:** ^1^Institute of Clinical Medical Science, China Medical University, Taichung, Taiwan; ^2^Department of Psychiatry and Brain Disease Research Center, China Medical University Hospital, Taichung, Taiwan; ^3^An Nan Hospital, China Medical University, Tainan, Taiwan; ^4^Biostatistical Consulting Laboratory, Department of Speech Language Pathology and Audiology, National Taipei University of Nursing and Health Sciences, Taipei, Taiwan; ^5^Department of Psychiatry, Taitung MacKay Memorial Hospital, Taitung, Taiwan; ^6^Department of Medicine, Mackay Medical College, New Taipei, Taiwan; ^7^Department of Psychiatry, Tzu-Chi General Hospital, Hualien, Taiwan; ^8^Institute of Medical Sciences, Tzu Chi University, Hualien, Taiwan

**Keywords:** theta-burst stimulation, transcranial magnetic stimulation, post-traumatic stress disorder, depression, dorsolateral prefrontal cortex

## Abstract

**Background:**

Theta-burst transcranial magnetic stimulation has demonstrated promising effectiveness as treatment for post-traumatic stress disorder (PTSD) and depression. However, the effect of accelerated theta-burst stimulation (TBS) in comorbid with PTSD and depression remains unknown.

**Case presentation:**

We report a case of a 25-year-old woman with PTSD and depression whose symptoms markedly improved after continuous TBS of the right dorsolateral prefrontal cortex (DLPFC) and intermittent TBS (iTBS) over the left DLPFC, and then with 20 min break before the 2nd iTBS session.

**Conclusions:**

Accelerated TBS over the bilateral DLPFC may improve severe PTSD and depression. Accelerated TBS may have more improvement of depression symptoms than PTSD symptoms. Further trials are warranted to investigate the effect and safety for patients with complicated PTSD and depression.

## Introduction

Post-traumatic stress disorder (PTSD) is a common psychiatric disorder characterized by intrusive thoughts or flashbacks, avoidance of trauma-related stimuli, negative alterations in mood and thought, and hyperarousal. An estimated 6–7% of adults in the general population of the United States will experience PTSD during their lifetime (1). Moreover, several studies have indicated that the prevalence of major depressive disorders in patients with PTSD ranges from 25 to 50% (2, 3); the available data support the existence of a shared neurobiology between major depressive disorder and PTSD (4). Although evidence-based PTSD treatments, including psychotherapy and pharmacology, have been demonstrated to be effective at alleviating symptoms (5, 6), standard pharmacological treatments and psychotherapy often produce only a partial response, indicating that a considerable proportion of patients are treatment resistant (7).

Theta-burst stimulation (TBS) is a novel transcranial magnetic stimulation (TMS) treatment which short bursts of high frequency (50 Hz) stimulation are repeated at 5 Hz (200 ms interval). TBS including intermittent TBS (iTBS) and continuous TBS (cTBS) can rapidly induce synaptic plasticity (8). Pilot studies have reported the effects of TBS on patients with PTSD (9–12). For example, Philip et al. enrolled 50 veterans with PTSD who received 10 days of sham–controlled iTBS (1,800 pulses/day) over right dorsolateral prefrontal cortex (DLPFC), followed by 10 unblinded sessions. After 2 weeks of treatment, the iTBS group demonstrated significant improvement with respect to social and occupational function (Cohen's d = 0.39; *p* = 0.04) (9). Moreover, previous studies have showed that TBS treatment has promising effect in patients with depression (13). Combined cTBS/iTBS of bilateral DLPFC have revealed a superior effect compared to unilateral therapy on depression (14, 15). In addition to regular TBS (600 pulses), new protocols including prolonged TBS and accelerated TBS have been investigated. Prolonged TBS generally refers to x2-3 more pulses per session (1,200–1,800 pulses), which in some studies have been shown to have the reverse effect on cortical excitability (16). Accelerated TBS generally refers to multiple sessions per day (more than one daily session) (13). Pilot studies have found that accelerated TBS with interval may enhance after effect (16, 17), and lea d to greater alleviation of depression. However, the effect of accelerated theta-burst stimulation (TBS) in patients with PTSD and depression remains unclear.

Previous studies have demonstrated antidepressant effects of bilateral TBS (left sided iTBS + right sided cTBS) in the treatment of major depression (18–20). However, due to that patient's urgent clinical status, we needed to improve her condition rapidly. There have been many researches regarding the use of iTBS on the left DLPFC with 20–30 intervals in the treatment of depression (21, 22). To date, there have been no studies using accelerated TBS in the treatment of PTSD combined with depression. Thus, in the case, we chose to add additional iTBS 600 pulses treatment with 20 min interval to stimulate left DLPFC.

Herein, we report a case of a 25-year-old woman with severe PTSD and depression whose symptoms markedly improved after receiving accelerated TBS to the bilateral DLPFC.

## Case report

A 25-year-old woman was brought to our psychiatric clinic for severe PTSD and depression with suicidal ideation. Her depressive symptoms developed at the age of 20 after she was verbally abused by a teacher and failed an important project. In addition to a depressed mood, she experienced a loss of interest, feelings of worthlessness, psychomotor retardation, and suicidal ideations. Her family brought her to a psychiatric clinic, where major depressive disorder (MDD) was diagnosed. She began taking escitalopram (10 mg/d increasing to 20 mg/d in 1 month) and receiving psychotherapy. Her symptoms of MDD gradually improved later and she could graduate from college on schedule.

However, in August 2020, she experienced unexpected verbal violence and threats from a colleague at her place of work. Her colleague often consumed alcohol, slammed doors, and swore at her, which caused her to feel stressed. She developed hyperarousal, re-experience (flashbacks and nightmares), avoidance (avoiding the office and her colleague), and transient auditory hallucinations (an unknown voice criticizing her), which persisted for more than a month. She then experienced a relapse of depression with suicidal ideation. PTSD and major depressive disorder were diagnosed according to the diagnostic and statistical manual of mental disorders-5 (DSM-5) criteria (23, 24). Medications including escitalopram 20 mg per day (4 weeks), duloxetine 60 mg per day (4 weeks), venlafaxine 150 mg per day (4 weeks), and a combination of 60 mg duloxetine and 5 mg aripiprazole daily (4 weeks) were subsequentially used. She also received psychotherapy for 4 weeks. However, she did not respond well to these medications and suffered from the drug side effects including dizziness, nausea, and weight gain. She refused additional antidepressant treatment and kept venlafaxine 150 mg per day. Therefore, she was referred to our brain stimulation center for an rTMS consultation. Her family has no history of MDD or PTSD.

We used the Davidson Trauma Scale (DTS) (25) to evaluate the severity of her PTSD. The DTS yields a frequency score (ranging from 0 to 68). The Beck Depression Inventory (BDI) (26) was used to evaluate the severity of her depression. The Beck Anxiety Inventory (BAI) was used to measure the severity of her anxiety symptoms (27). The BDI and BAI yields a frequency score (both ranging from 0 to 63). The severity of PTSD, depression, and anxiety increases with the scores. Higher scores mean worse PTSD, depression, and anxiety. She scored 62, 52, and 34 points on the DTS, BDI, and BAI, respectively.

**Figure 1:**
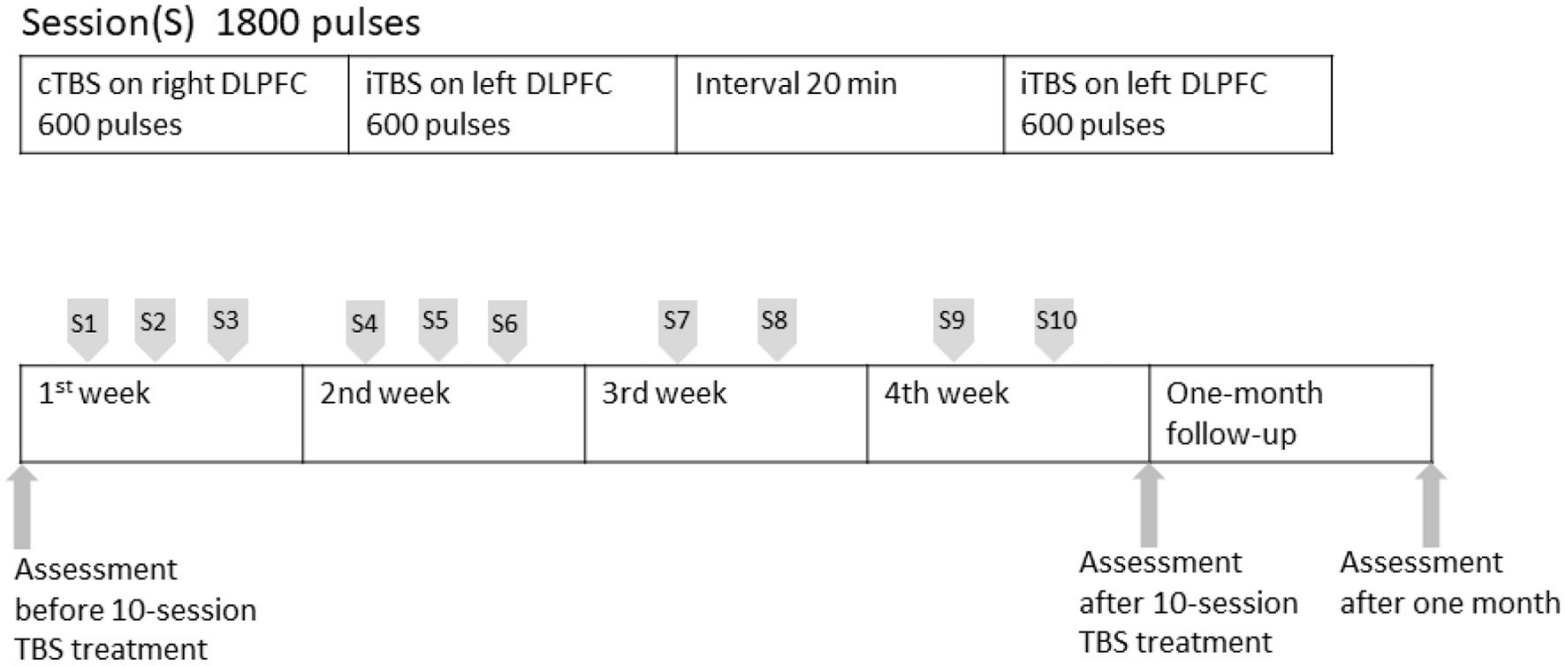
TBS procedure timeline.

After the patient provided signed informed consent, we adopted a new rTMS treatment, theta-burst stimulation (TBS), of the bilateral DLPFC with an Apollo TMS Therapy stimulator (MAG and More, Munich, Germany) equipped with a figure-of-eight coil. First, we applied cTBS with 20 trains of 10 bursts (short bursts of 3 stimuli at 50 Hz, repeated at 5 Hz), with 600 pulses for 40 s at 80% RMT, targeting the right DLPFC. Second, we applied iTBS with 20 trains of 10 bursts (short bursts of 3 stimuli at 50 Hz, repeated at 5 Hz) given at 8 s intervals at 600 pulses for 200 s at 80% RMT, targeting the left DLPFC. After a 20-min intersessional rest period, we performed a second round of iTBS for 200 s, targeting the left DLPFC. Our design consists of 3 sessions, first two (cTBC, iTBS) being sequential and then with 20 min break before the 2nd iTBS session. The patient received 1,800 pulses in each treatment and total 18,000 pulses in the whole treatment course. The coil localization was based on the algorithm developed by Beam et al. (28). The Beam-F4 position was used for the right DLPFC and the Beam-F3 position was used for the left DLPFC (13). Because of her daily work and the COVID-19 pandemic, she cannot visit our department every day. The subsequent stimulation sessions were performed two to three times per week. We completed a total of 10-day (30 sessions) within 4 weeks (Figure 1).

The patient's PTSD and depressive symptoms gradually improved. After TBS treatment course, her symptoms improved (scores of 42, 7, and 4 on the DTS, BDI, and BAI, respectively) (Table 1). For the DTS scale assessment, symptom item 14 (increased irritability) and 15 (concentration difficulties) improved the most, from 4 to 1. The side effects of the treatment, such as dizziness or headache, were transient and disappeared after the TBS treatment finished.

**Table 1:** Clinical outcomes during the TBS treatment.

**Symptoms**	**Before TBS treatment**	**After TBS treatment**	**One month follow**
DTS	62	42	30
BDI	52	7	6
BAI	34	4	4

BAI, Beck Anxiety Inventory; BDI, Beck Depression Inventory; DTS, Davidson Trauma Scale; TBS, theta-burst stimulation.

## Discussion

To the best of our knowledge, this is the first reported case of application of accelerated TBS over the bilateral DLPFC in a patient with PTSD and MDD. Our patient demonstrated significant clinical improvements after TBS treatment sessions without severe adverse effects. Second, accelerated TBS may have more improvement of depression symptoms than PTSD symptoms. Third, we reviewed the current TBS studies in PTSD (Table 2).

**Table 2:** Main characteristics of TBS studies in PTSD.

**Study**	**Sample size**	**PTSD** **outcome measure**	**Depression outcome measure**	**Mean** **age**	**Brain target**	**Stimulation** **frequency**	**Settings**	**Research** **design**
Woodside et al. (12)	14	PCL-C Pre 54.29 ± 19.34 Post 24.86 ± 17.43 (Improvement 54.2%)	Nil	39.8	DMPFC	rTMS (10 and 20 Hz) and iTBS	120% RMT Total Pulses >36,000 Sessions 20–30	Open-label
Philip et al. (9)	50	CAPS-5 Active 47.9–38.6 (Improvement 19.4%) Sham 47.4–39.4 PCL- 5 Active 49.4–35.5 (Improvement 28.1%) Sham 50.0–39.4	IDSSR Active 42.8–31.3 (Improvement 26.9%) Sham 39.2–33.5	48	Right DLPFC	Right sided iTBS	80 % AMT Total Pulses 15,000–36,000 Sessions>19	RCT
Nursey et al. (10)	8	CAPS-5 Pre 47.38 ± 6.16 Post 37.00 ± 9.04 (Improvement 21.9%)	HAM-D Pre 21.25 ± 2.82 Post 16.00 ± 6.08 (Improvement 24.7%)	37.5	Bilateral DLPFC	Bilateral iTBS	120% RMT Total Pulses 15,000–36,000 Sessions 20	Open-label
Vaithianathan et al. (11)	8	PCL-5 Pre 43.5 ± 18.57 Post 29.8 ± 19.3 (Improvement 31.5%)	HAMD-17 Pre 23.0 ± 4.4 Post 12.9 ± 5.7 (Improvement 43.9%)	48.75	Bilateral DLPFC	right sided cTBS left sided iTBS	80 to 120% RMT Total Pulses ≥ 12,000 Sessions ≥ 10	Open-label cohort observation

AMT, Active motor threshold; TMS, Transcranial Magnetic Stimulation; DLPFC, Dorsolateral Prefrontal Cortex; DMPFC, Dorsomedial prefrontal cortex; CAPS, Clinician Administered Posttraumatic Stress Scale; PCL, Post-traumatic Stress Disorder Symptom Checklist; RCT, Randomized Controlled Trial; RMT, Resting motor threshold; PCL-5, The PTSD Checklist for DSM-5; PCL-C, Post-traumatic Stress Disorder Symptom Checklist Civilian Version; IDSSR, Inventory of Depressive Symptomatology Self-Report; TBS, theta burst stimulation; cTBS, continuous TBS; iTBS, intermittent TBS.

Our findings agreed with previous TBS studies in PTSD (9–12) (Table 2). Two studies used PCL, one study used CAPS, and one study used both to evaluate the severity of PTSD. All trials reported improvement in the severity of PTSD. Three of four studies were open-label (10–12), while Philip's study was RCT (9). Philip et al. enrolled 50 veterans with PTSD who received 10 days of sham–controlled iTBS (1,800 pulses/day) over right dorsolateral prefrontal cortex (DLPFC), followed by 10 unblinded sessions. After 2 weeks of treatment, the CAPS scores improved from 47.9 to 38.6, and PCL from 49.4 to 35.5 (9).

In our case, the patient had a history of major depressive disorder prior to PTSD. Different TMS protocols have been shown to be clinically effective, but the response rate varies. Depressed patients may have comorbidities such as substance use disorder, generalized anxiety disorder, or PTSD. Personalized rTMS treatment for patients with major depressive disorder and psychiatric comorbidities are suggested (29). Our results showed more improvement of depression than PTSD. The scores of DTS improved from 62 to 42 (improvement 32.5%) while the scores of BDI improved from 52 to 7 (improvement 86.5%). Our findings agreed with previous two studies (10, 11). The improvement of depression was better than the improvement of PTSD (24.7 vs. 21.9% in Nursey' study and 43.9 vs. 31.5% in Vaithianathan's study). In Philip's study, the improvement of depression in active TBS group was better than the improvement of PTSD (IDSSR 26.9% vs. CAPS 19.4%), but was not in another scale (IDSSR 26.9% vs. PCL 28.1%).

Several reasons may have contributed to the difference in depression between the results in Philip et al. and other studies. First, Philip and his colleagues used IDSSR instead of standard depression measures like HAMD. Second, the target was right DLPFC in Philip's study while two studies targeted bilateral DLPFC. Multisite stimulation such as bilateral TBS treatment may lead to a larger response than single-site stimulation (20). In our study, we targeted the bilateral DLPFC, whereas Philip et al. targeted only the right DLPFC. Previous studies have shown that combined cTBS/iTBS of bilateral DLPFC may have a superior effect compared to unilateral therapy on depression (14, 15). A randomized sham-controlled study enrolling 60 treatment-refractory patients with recurrent major depressive disorder revealed that the mean antidepressant effect was highest in a combination of iTBS and cTBS (14). A three-week treatment of iTBS over left DLPFC and cTBS of the right DLPFC (DLPFC) in patients with treatment-resistant depression (TRD) reported that significantly reduced functional connectivity between the left stimulation target and bilateral anterior insula (30). Third, our patient underwent accelerated iTBS over the left DLPFC with a 20 min interval, whereas the patients in the study by Philip et al. received iTBS over the right DLPFC without interval.

Several physiopathogenic hypotheses may support the effectiveness of TBS over bilateral DLPFC. First, previous animal and huma studies have found that the hyperactivity of the amygdala and dorsal anterior cingulate cortex can promote fear responses and associated with PTSD. The hypoactivity of ventromedial and DLPFC is involved in the suppression of fear responses (31–33). TBS over bilateral DLPFC suppress the fear response. Second, the improvement in PTSD could be associated with the improvement in depression. Depression may play a role in the development and aggravation of PTSD. TBS may improve the severity of depression-associated PTSD (34). Third, cTBS treatment of the right DLPFC could serve as priming for stimulation of the left DLPFC (35). Therefore, the second stimulation targeting the left DLPFC can be enhanced by the priming stimulation over the right DLPFC.

However, because our case lacked a placebo control group, the results should be interpreted with caution. This case report has several limitations. First, only one patient was evaluated. Further studies with larger samples and randomized designs are required to verify the effects of accelerated TBS over the bilateral DLPFC. Second, gold-standard measure like the Clinician-Administered PTSD Scale for DSM-5 (CAPS-5) (36, 37) was not used in this case report because the lack of the Taiwanese version of CAPS-5. We will launch a Taiwanese language validation study of CAPS-5 in the near future.

## Conclusion

Accelerated TBS over the bilateral DLPFC may improve severe PTSD and depression. Besides, accelerated TBS may have more improvement of depression symptoms than PTSD symptoms. Future studies with larger sample sizes and randomized, double-blind designs and placebo-controlled trials are warranted to verify our findings.

## Data availability statement

The original contributions presented in the study are included in the article/supplementary material, further inquiries can be directed to the corresponding author.

## Ethics statement

Written informed consent was obtained from the patient for publication of this case report.

## Author contributions

C-HC drafted the initial manuscript. S-JC and C-YL provided expert opinions. H-CT critically reviewed the draft of manuscript. C-HC and H-CT approved the final submitted version manuscript. All authors contributed to the article and approved the submitted version.
